# Genomic Characterization and Virulence Determinants of *Staphylococcus aureus* Clinical Isolates from Pneumonia Patients in Karaganda, Kazakhstan

**DOI:** 10.3390/antibiotics15050431

**Published:** 2026-04-25

**Authors:** Shynggys Orkara, Vitaliy Strochkov, Alyona Lavrinenko, Nurlan Sandybayev

**Affiliations:** 1 Kazakhstan-Japan Innovation Centre, Kazakh National Agrarian Research University, Almaty 050010, Kazakhstan; vitaliy.strochkov@kaznaru.edu.kz; 2 Scientific Research Laboratory, Karaganda Medical University, Karaganda 100000, Kazakhstan; lavrinenko@qmu.kz

**Keywords:** *Staphylococcus aureus*, pneumonia, antimicrobial resistance, MRSA, whole-genome sequencing, MLST, virulence factors

## Abstract

**Background/Objectives**: *Staphylococcus aureus*, particularly methicillin-resistant strains, is a leading cause of severe pneumonia. Understanding local molecular epidemiology, including virulence gene profiles and antimicrobial resistance (AMR) mechanisms, is crucial for effective infection control. This pilot study aimed to characterize *S. aureus* isolates from pneumonia patients in Karaganda, Kazakhstan. **Methods**: We collected 48 respiratory samples from patients with pneumonia across three medical institutions. Bacterial identification was performed using MALDI-TOF MS. Antimicrobial susceptibility testing (AST) was carried out using European Committee on Antimicrobial Susceptibility Testing (EUCAST) guidelines. Whole-genome sequencing of *S. aureus* isolates was conducted on an Ion Torrent S5 platform. Genomic analysis included multilocus sequence typing (MLST), identification of virulence and AMR genes, and phylogenetic reconstruction. **Results**: *S. aureus* was identified in 14.6% (*n* = 7) of pneumonia cases included in this study. All isolates (100%, *n* = 7) were phenotypically resistant to benzylpenicillin. The *mecA* gene was detected in 57.1% of isolates (*n* = 4), while phenotypic resistance to methicillin was observed in 28.6% (*n* = 2) of the isolates. Resistance to azithromycin (57.1%, *n* = 4) and levofloxacin (42.9%, *n* = 3) was observed among the isolates. Two isolates (28.6%) were multidrug-resistant (MDR). Genomic analysis revealed the prevalence of the ST22 clone (57.1%, *n* = 4) in the studied cohort. Other sequence types were ST97, ST8, and ST45 (14.3% each). Phylogenetic analysis showed clustering consistent with MLST profiles. All isolates carried a conserved core virulence arsenal, including hemolysin (*hla*, *hlg*), biofilm-forming genes (*icaADBC*), immune evasion genes (*sak*, *scn*), and iron acquisition genes (*isd*). The Panton–Valentine leukocidin (PVL) genes were detected in three isolates. AMR gene analysis revealed the ubiquitous presence of *mepA* and tetracycline efflux pump genes, along with regulatory genes (*arlRS*, *mepR*, *mgrA*). The *blaZ* and *ermA* genes were not detected despite high phenotypic resistance to penicillin and macrolides. **Conclusions**: This study reports the identification of the virulent and resistant ST22 *S. aureus* clone in pneumonia cases in Karaganda, Kazakhstan. The discordance between phenotypic and genotypic AMR profiles underscores the necessity for integrated diagnostic approaches.

## 1. Introduction

*Staphylococcus aureus* is a clinically significant opportunistic pathogen responsible for a wide spectrum of diseases, ranging from superficial skin and soft tissue infections to life-threatening conditions such as endocarditis, osteomyelitis, sepsis, and pneumonia [1,2]. As a leading cause of both nosocomial and community-acquired infections, it contributed to millions of cases of invasive diseases worldwide annually [3].

Staphylococcal pneumonia is of particular clinical importance, as it is associated with severe disease in hospital settings and with epidemic outbreaks in the community [4]. In hospitalized patients, especially those receiving mechanical ventilation, mortality rates may reach up to 55% [5], whereas in community-acquired pneumonia among children, reported case-fatality rates range from 0.8% to 7% [6,7]. Despite the successes of modern medicine, *S. aureus* remains the leading cause of bloodstream infections and pneumonia, characterized by significant mortality [8,9,10].

The global spread of methicillin-resistant *S. aureus* (MRSA) further amplifies this challenge. In several regions, MRSA accounts for up to 25–50% of clinical isolates, substantially limiting therapeutic options [11]. At the same time, increasing attention has been directed toward methicillin-susceptible *S. aureus* (MSSA). Certain MSSA lineages, such as sequence type ST398, exhibit high virulence and are capable of causing severe and sometimes fatal infections [12]. Moreover, experimental models have demonstrated that some MSSA strains may be more pathogenic than MRSA, for example, in a *Caenorhabditis elegans* infection model, the mean survival time was three days for MSSA compared with six days for MRSA [13]. Epidemiological studies of MSSA in West Australia have also revealed considerable diversity in virulence determinants, including a high prevalence of MSCRAMM proteins and α-toxin in more than 90% of isolates [14].

The clinical landscape is further complicated by the presence of borderline oxacillin-resistant *S. aureus* (BORSA), which displays a methicillin-resistant phenotype in the absence of the *mecA* or *mecC* genes [15]. The proportion of such strains has been reported to range from 0.5% to 70% in different regions [16]. This phenomenon significantly complicates the diagnosis and epidemiological surveillance. The problem is that standard methods such as PCR for the *mecA* gene or the disk diffusion method with cefoxitin are not able to identify this phenotype [15]. In addition, 20–60% of respiratory infections in the world remain undiagnosed using conventional diagnostic tools [17]. In Kazakhstan, where a massive empirical prescription of antibiotics and a high incidence of bacterial co-infections occurred during the COVID-19 pandemic [18], the lack of in-depth genomic monitoring creates a gap not only in understanding the structure of antibiotic resistance of circulating strains, but also in assessing their virulent potential [15].

The pathogenicity of *S. aureus* is driven by a diverse arsenal of virulence factors. These include pore-forming toxins like α-hemolysin, immune evasion molecules (e.g., Protein A), adhesins, and cytotoxins targeting host immune cells [3]. Panton–Valentine leukocidin (PVL) is a key determinant associated with severe necrotizing pneumonia and poorer clinical outcomes [19,20]. Furthermore, mechanisms such as biofilm formation and the coagulation of host plasma (mediated by coagulase, von Willebrand factor-binding protein, and fibronectin-binding proteins) facilitate immune evasion and systemic dissemination [10]. Critically, these virulence determinants often co-exist with AMR mechanisms, complicating treatment and worsening prognoses.

Taken together, the combination of diverse virulence factors and antimicrobial resistance mechanisms underscores the need for a comprehensive approach to studying the molecular basis of *S. aureus* infections. Whole-genome sequencing (WGS) has emerged as a powerful tool for comprehensive pathogen characterization, enabling high-resolution analysis of clonal structure, virulence gene profiles, and resistance determinants beyond the capabilities of conventional methods [21,22].

Therefore, this study aimed to perform an integrated phenotypic and genomic characterization of *S. aureus* isolates from pneumonia patients in Karaganda, Kazakhstan. Using WGS, we sought to determine the predominant sequence types (STs), elucidate their virulence gene repertoires, and decipher the genetic basis of observed antimicrobial resistance profiles. The findings are intended to provide critical baseline data for enhancing local epidemiological surveillance and informing targeted therapeutic and control strategies.

## 2. Results

### 2.1. Sample Collection

From a total of 48 sputum samples collected from adult patients with community-acquired pneumonia, seven (14.6%) yielded *S. aureus* upon culture and were confirmed by Matrix-Assisted Laser Desorption/Ionization Time-of-Flight Mass Spectrometry (MALDI-TOF MS). The clinical and demographic characteristics of these patients are summarized in Table 1. Isolates were obtained from two of the three participating institutions: the Hematology Center (HEM, *n* = 3) and the University Clinic of Karaganda Medical University (UNC, *n* = 4). Within these institutions, isolates originated from the Hematology (HO, *n* = 3) and Surgery (SUR, *n* = 3) departments, with one additional isolate from the Pulmonology (PUL) department. Clinical outcome data indicated that all patients were discharged following treatment.

### 2.2. Phenotypic Characterization of Antimicrobial Resistance in S. aureus

**Figure 1:**
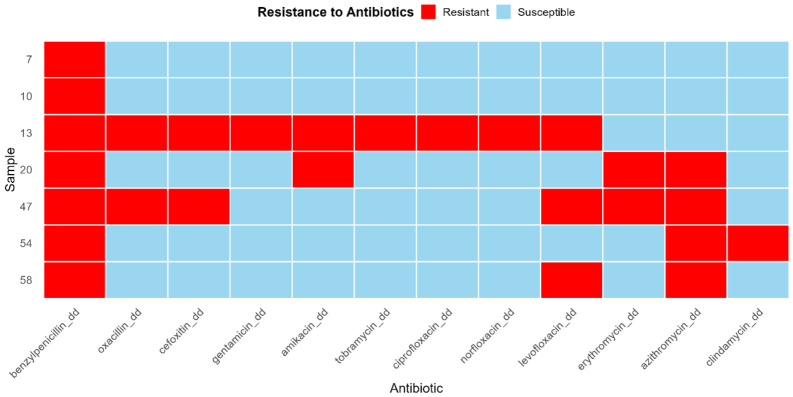
Phenotypic antimicrobial resistance profiles of *S. aureus* isolates from pneumonia patients in Kazakhstan. Resistance was determined by disk diffusion according to EUCAST v11.0 guidelines. The heatmap displays the susceptibility result for each isolate–antibiotic pair: red indicates a resistant (R) phenotype, and blue indicates a susceptible (S) phenotype.

All seven *S. aureus* isolates (100%) were resistant to benzylpenicillin. Based on cefoxitin disk diffusion, two isolates (28.6%) were phenotypically classified as methicillin-resistant *S. aureus*. The overall resistance profiles revealed considerable diversity, ranging from resistance to a single antibiotic class (penicillin) to multidrug resistance (MDR, defined as non-susceptibility to 3 antimicrobial classes). Two isolates (28.6%) were classified as MDR: isolate No. 13 was resistant to nine antibiotics across three classes, and isolate No. 47 was resistant to six antibiotics across three classes. Beyond penicillin, the most prevalent resistances were to the macrolide azithromycin (57.1%, *n* = 4) and the fluoroquinolone levofloxacin (42.9%, *n* = 3). Lower resistance rates were observed for erythromycin (28.6%, *n* = 2), amikacin (28.6%, *n* = 2), gentamicin (14.3%, *n* = 1), norfloxacin (14.3%, *n* = 1), and ciprofloxacin (14.3%, *n* = 1). The complete susceptibility profiles are visualized in the resistance heatmap (Figure 1).

### 2.3. Sequencing Quality and Genome Assembly

Whole-genome sequencing of the seven *S. aureus* isolates yielded high-quality data suitable for downstream analysis.

Genome assembly of the seven *S. aureus* isolates resulted in genomes with an average total length of 2,593,459 bp (range: 2,544,746–2,695,876 bp). The mean GC content was 32.85% (range: 32.70–32.99%), which is consistent with the typical genomic characteristics of *S. aureus*.

The assemblies consisted of an average of 137 contigs per genome (range: 102–161). Assembly continuity, assessed by the N50 metric, showed a mean value of 43,275 bp, ranging from 36,549 to 74,430 bp. Detailed assembly and sequencing metrics are provided in Supplementary Table S1.

For phylogenetic contextualization, six additional genomes were included in the analysis: three contemporary isolates from other infection sources in Kazakhstan (OTT1-2021 (GenBank ID: CP082813.1), WND1-2021 (NZ_CP082815.1), OTT1-2022 (CP102945)) and 2 reference strains (NCTC 8325 (CP000253), MOK063 (CP029629.1)), and *Staphylococcus epidermidis* (ATCC 14990 (GCF_006094375.1) as an outgroup.

### 2.4. MLST and Clonal Diversity

To characterize clonal diversity, multilocus sequence typing (MLST) was performed using the PubMLST online database (Pasteur scheme). Among the clinical isolates from pneumonia patients, the predominant clone was ST22 (four of seven isolates, 57.1%), which belongs to the epidemiologically significant clonal complex CC22. The remaining isolates were ST97 (14.3%, *n* = 1), ST8 (14.3%, *n* = 1), and ST45 (14.3%, *n* = 1), corresponding to clonal complexes CC97, CC8, and CC45, respectively. Comparative analysis with isolates from other sources collected in 2021–2022 revealed additional diversity: a wound infection isolate (WND1_2021) was assigned to ST30 (CC30), while ear infection isolates were assigned to ST508 (OTT1_2021) and ST78 (OTT1_2022).

**Figure 2:**
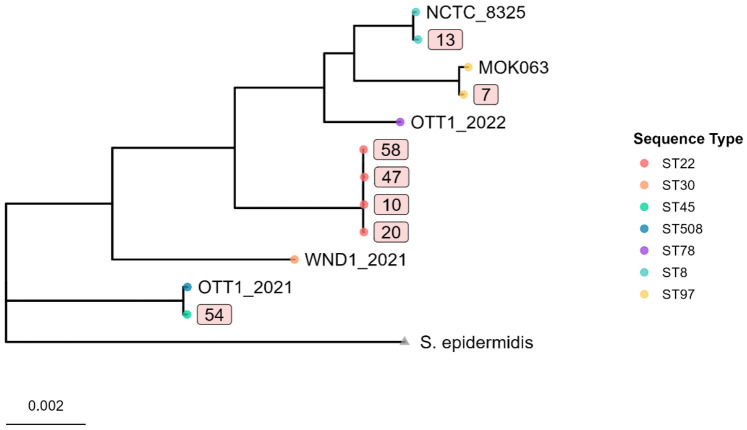
Core genome maximum-likelihood phylogeny of *S. aureus* isolates from Kazakhstan, rooted with *S. epidermidis* ATCC 14990 as an outgroup. The tree includes seven pneumonia isolates from this study (rectangles labels) and five previously sequenced isolates from other clinical sources. Branches are colored by sequence type (ST).

A maximum-likelihood phylogenetic tree, constructed from a core genome alignment of the seven study isolates and five additional *S. aureus* genomes from Kazakhstan, and *S. epidermidis* (ATCC 14990), confirmed the MLST-based classification. Isolates clustered into distinct, well-supported clades according to their sequence type (Figure 2).

Notably, the four ST22 pneumonia isolates formed a tight, monophyletic cluster.

### 2.5. Antimicrobial Resistance (AMR) Genes

Analysis of the seven pneumonia-associated *S. aureus* genomes revealed a diverse profile of AMR genes, with 8 to 11 genes detected per isolate (Table S2). A core set of genes was ubiquitous (*n* = 7, 100%), including the multidrug efflux pump gene *mepA*, the tetracycline resistance gene *tet*(38), and the global regulatory genes *arlRS*, *mepR*, and *mgrA*.

Genes essential for intrinsic β-lactam resistance (*pbp2*, *femA*) were detected in all genomes. Notably, the canonical methicillin resistance gene *mecA* was identified in four of the seven pneumonia isolates (57.1%).

**Figure 3:**
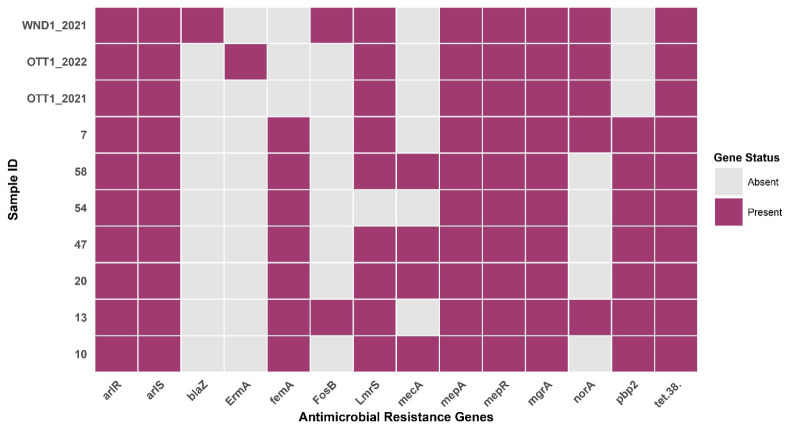
Distribution of AMR genes among *S. aureus* isolates. The heat map shows the presence (purple) or absence (gray) AMR genes in 10 studied isolates.

Analysis extended to the three additional Kazakh isolates from other infection sources (OTT1_2021, WND1_2021, OTT1_2022) revealed a broader AMR gene pool. The *blaZ* gene was identified in the wound isolate WND1_2021 (ST30), and *ermA* was found in the ear isolate OTT1_2022 (ST78)—genes absent in the pneumonia cohort. The distribution of all key AMR genes across the combined set of ten isolates is summarized in the heatmap (Figure 3).

The *lmrS* gene, encoding another multidrug efflux pump, was detected in 90% of isolates (*n* = 9), being absent only in isolate No. 54. Among genes conferring resistance to specific antibiotic classes, *norA* (a fluoroquinolone efflux pump) was the most prevalent, present in half of the isolates (50%, *n* = 5). Genes mediating resistance to fosfomycin (*FosB*) and macrolides (*ermA*), as well as the beta-lactamase gene (*blaZ*), were less frequent. *FosB* was identified in two isolates (20%).

Importantly, the *mecA* gene, a hallmark of methicillin-resistant *S. aureus*, was identified in four clinical isolates, Nos. 10, 20, 47, and 58 (57.1%, *n* = 4), indicating the circulation of genetically confirmed MRSA strains within the studied cohort. The presence of *mecA* was accompanied by the detection of *pbp2* and *femA*, genes involved in peptidoglycan synthesis and expression of high-level methicillin resistance.

In addition, oxacillin-resistant isolate No. 13 lacked *mecA* but exhibited phenotypic resistance to oxacillin, consistent with a borderline oxacillin-resistant *S. aureus* phenotype.

In contrast, isolate No. 47 was characterized by the simultaneous presence of the *mecA* gene and phenotypic resistance to oxacillin, which confirms that it belongs to methicillin-resistant *S. aureus*. Interestingly, the remaining isolates carrying the *mecA* gene were phenotypically sensitive to oxacillin and cefoxitin, representing the so-called cryptic or hidden MRSA genotypes. This mismatch between genotype and phenotype indicates the presence of undetected or poorly expressed *mecA*, which may lead to an underestimation of the prevalence of MRSA if relying solely on phenotypic testing.

Thus, the studied population comprised classical MRSA, *mecA*-negative BORSA strains, as well as cryptic *mecA*-positive but phenotypically susceptible isolates, highlighting the complexity of β-lactam resistance mechanisms in circulating clinical *S. aureus* strains.

### 2.6. Virulence Genes

Similar to the resistance analysis, the virulence factors were characterized for the same group of 10 clinical isolates. The number of identified virulence genes per genome ranged from 47 to 67 (Table S3). The most conserved genes were those encoding key factors associated with hemolysis, biofilm formation, immune evasion, and iron acquisition, present in the majority of isolates (90–100%): *adsA*, *cap8* (*A–G*, *L–P*), *geh*, *hld*, *hlgA/B/C*, *hly/hla*, *icaA–D*, *isdA–G*, *lip*, *sak*, *scn*, *sspA–C*, and *srtB*.

When virulence profiles were compared according to antimicrobial resistance phenotype, clear differences were observed between MRSA, BORSA and MSSA groups.

The four *mecA*-positive MRSA isolates exhibited a highly conserved virulence core but lacked several adhesins and immune evasion genes detected in BORSA and MSSA isolates, indicating a relatively homogeneous virulence profile among MRSA strains.

In 40–70% of isolates, additional genes belonging to the MSCRAMM family (microbial surface components recognizing adhesive matrix molecules) and other adhesins were detected, including *clfA*, *clfB*, *fnbA*, *sdrC*, *sdrD*, *sdrE*, *spa*, *map*, and *coa*.

The BORSA isolate No. 13 demonstrated a diverse set of virulence, harboring *spa*, *sdrD*, *vWbp*, as well as multiple MSCRAMM genes, resulting in the highest total number of virulence determinants among all analyzed genomes.

Genes encoding components of the type VII secretory system (esaC, essC, esxB) and additional proteases (hysA) were found only in MSSA BORSA isolates, whereas these genes were absent in other MRSA strains.

**Figure 4:**
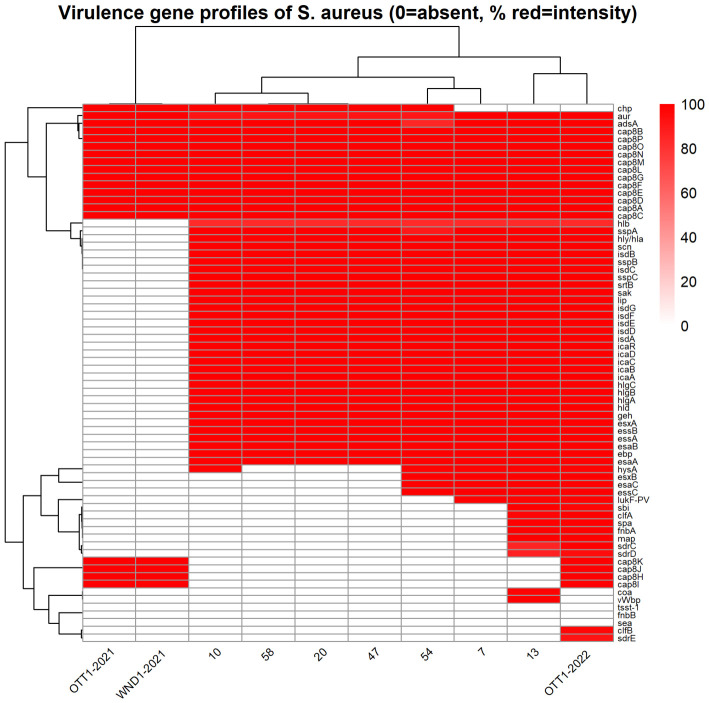
Heatmap of virulence gene occurrence across *S. aureus* isolates.

The pore–forming cytotoxin leukocidin Panton–Valentine (LukF-PV) was detected sporadically and was present only in three isolates No. 7, 13 and one comparative (OTT1-2022). The overall distribution of virulence genes is summarized in Figure 4.

Within our small study cohort, MRSA isolates tended to exhibit a more limited virulence gene set, whereas BORSA and MSSA isolates showed greater heterogeneity and carried a broader range of adhesins and immune evasion factors. This observation points to the possibility that high virulence potential may not be exclusive to MRSA and can be present in *mecA*-negative strains in this specific clinical setting.

## 3. Discussion

Whole-genome sequencing of *S. aureus* isolates from patients with pneumonia in Karaganda, Kazakhstan provided preliminary insights into the genomic diversity of AMR. The epidemiological data for Kazakhstan indicate a specific and variable MRSA landscape, which is characterized by a relatively low but regionally variable prevalence compared to neighboring countries [23,24]. Similar trends in genomic diversity and the formation of therapy-resistant clones have previously been described for other nosocomial pathogens in the region [25]. These findings highlight the importance of monitoring local resistance patterns.

In our study, two isolates were identified that were phenotypically resistant to beta-lactams, No. 47 and No. 13 (28.6%, *n* = 2). Simultaneously, only isolate No. 47 was a carrier of the *mecA* gene and is classified as classic MRSA, whereas isolate No. 13 demonstrated resistance to oxacillin and cefoxitin in the absence of *mecA*, corresponding to the BORSA phenotype.

The discovery of three isolates (Nos. 10, 20, and 58) carrying the *mecA* gene, but remaining phenotypically sensitive to oxacillin and cefoxitin, is of particular interest. This phenotype, known as cryptic MRSA (oxacillin- and cefoxitin-susceptible *mecA*-positive *S. aureus*), poses a serious diagnostic problem, because such strains are mistakenly classified as MSSA in routine practice [26]. According to modern concepts, *mecA*-positive but phenotypically sensitive isolates can maintain latent resistance potential and induce PBP2a expression under the influence of antibacterial pressure, leading to the formation of a true MRSA phenotype [27]. In this regard, the CLSI and EUCAST recommendations prescribe that all *mecA*-positive strains should be considered as resistant to beta-lactam antibiotics, regardless of the results of phenotypic tests, because their therapy with beta-lactams is associated with the risk of clinical inefficiency [28,29].

The mechanisms of formation of cryptic MRSA can be different: from mutations in the regulatory region of the *mecA* gene, leading to a decrease in its expression, to insertions or deletions in tandem repeats within the gene itself, causing a shift in the reading frame and the appearance of a premature stop codon [30,31].

Conversely, we identified one *mecA*-negative isolate (No. 13, ST8) with phenotypic resistance, consistent with a BORSA profile. BORSA mechanisms often involve β-lactamase hyperproduction, mutations in native penicillin-binding proteins (PBPs), or cell wall thickening via pathways like *gdpP* inactivation [28,32]. Although the clinical relevance of BORSA is frequently underestimated, such isolates represent a tangible therapeutic threat, as infections can be severe and may not respond optimally to standard β-lactam therapy [32]. Notably, BORSA isolate No. 13 harbored the most comprehensive virulence gene profile, including *spa*, *sdrD*, *vWbp*, and multiple MSCRAMM-associated genes. The identification of both cryptic MRSA and BORSA in this cohort suggests the potential complexity of the local resistance landscape, suggesting that hidden resistance mechanisms may be more prevalent in the region than previously recognized through conventional methods. Previously, there was a relatively low prevalence of MRSA (for example, 3.2% in Astana, up to 12.5% in Karaganda) against the background of high rates in Russia (18–48%) or China (up to 72.8%) [23,24]. Traditional methods of phenotypic screening probably underestimate the true genetic potential of resistance [26]. Phenotypes similar to the cryptic MRSA described by us can be classified as MSSA, which underestimates MRSA indicators and masks a hidden threat. At the same time, BORSA isolates, which can cause severe infections, require a special therapeutic approach [26].

In our cohort, *femA* and *pbp2* genes were found in all major clinical isolates (n = 7), regardless of whether they belong to the MSSA, cryptic MRSA or BORSA phenotypes. Although these genes encode essential components involved in cell wall biosynthesis and may influence resistance levels [33,34], their specific contribution to the phenotypes observed in this study remains hypothetical. We hypothesize that reduced *femA* expression may limit resistance in MRSA isolates [35]. However, confirmation of this mechanism requires further transcriptomic analysis.

In our cohort, β-lactam resistance in *mecA*-negative isolates may be associated with alterations in endogenous PBPs [33]. Although mutations in *pbp2* are well-established mechanisms of β-lactam resistance in *mecA*-negative strains [36,37], the specific contribution of *pbp2* to the resistance observed in isolate No. 13 remains unclear, as sequencing or expression analysis of this gene was beyond the scope of our study.

The distribution of virulence and resistance genes in our isolates appears to align with the hypothesis of a fitness trade-off associated with the maintenance of the SCCmec cassette [38]. It is possible that the cryptic MRSA phenotype correlates with reduced metabolic costs, although this observation remains preliminary given the limited sample size (*n* = 7). A reduction in *mecA* expression could potentially preserve competitive growth and allow redistribution of cellular resources, accounting for the specific virulence profiles observed in these isolates [39].

In this context, the BORSA phenotype represented by isolate No. 13 could potentially reflect a different metabolic pattern. By not bearing the metabolic burden of SCCmec, this strain appears to have gained an evolutionary advantage, allowing resources to be allocated toward the maintenance and enhancement of virulence (a rich repertoire of adhesins and toxins). In this cohort, this strategy could potentially confer a selective advantage in specific ecological niches.

The detection of *arlRS*, *mgrA*, and *mepR* genes in all isolates, both clinical and comparative, suggests their potential involvement in adaptive responses to antibiotics. Dysregulation of the *ArlRS–MgrA* axis can alter β-lactam susceptibility and contribute to the development of BORSA-like phenotypes through cell wall thickening and reduced autolysis [40]. At the same time, *MgrA* regulates the expression of efflux pumps, including *norA* and *mepA*, which can enhance resistance to macrolides and fluoroquinolones [41]. These observations suggest that *S. aureus* resistance emerges through the integration of chromosomal mechanisms, regulatory networks, and selective antibiotic pressure.

The presence of the efflux pump genes *mepA* (100%) and *lmrS* (90%) provides insight into the high frequency of azithromycin resistance (57.1%) despite the absence of classical *erm* genes [42]. *MepA*, a member of the MATE (Multidrug and Toxic Compound Extrusion) family, is a known determinant of resistance to fluoroquinolones, tigecycline and other antimicrobial agents [43,44]. Its expression is negatively regulated by the repressor *MepR* [44,45]. The detection of *mepR* in all isolates, combined with the observed resistance phenotype, could indicate possible derepression of *MepA* in this cohort, potentially leading to increased MICs [43,44]. Thus, chromosomal efflux systems appear to constitute the basis of intrinsic macrolide resistance in this cohort [42].

The resistance profile observed in our isolates, particularly the absence of *blaZ* and *erm* genes, contrasts with reports from neighboring and Asian countries, where these determinants are frequently detected [46,47,48]. Even within Kazakhstan, the data are heterogeneous: in our study of clinical isolates, these genes were detected only in isolate WND1_2021, whereas studies on isolates from food products and livestock report considerably higher frequencies [49,50]. This discrepancy may reflects differences in selective pressures between clinical and veterinary environments. However, it is also necessary to consider the technical limitations of our fragmented genome assemblies (averaging 137 contigs). Nevertheless, the detection of sequence type ST97 in one clinical isolate, phylogenetically related to LA-MRSA, highlights the importance of resistance monitoring within a “One Health” framework and tracking potential interspecies transmission pathways [51].

In our isolates, we observed a pattern suggestive of segregation of the ess locus: while Module 1 was conserved across all strains, Module 2 genes were identified only in MSSA and BORSA isolates. This pattern may reflect a potential evolutionary trade-off or a lineage-specific genomic variation within our small cohort [52].

This observation warrants further investigation, as essC homologs have been identified in all examined *S. aureus* strains in global collections, including MRSA lineages such as USA300 (essC1 variant) and MRSA252 (essC3 variant) [52]. The apparent absence of Module 2 in our MRSA isolates requires cautious interpretation. While it could suggest a region-specific deletion, it is equally possible that this results from technical artifacts. Further analysis using long-read sequencing or PCR validation would be necessary to definitively confirm the loss of these genes.

We hypothesize that the distribution of these genes might be associated with inter-strain competition. Current evidence indicates that T7SS plays a key role in intraspecies competition by secreting nuclease toxins (e.g., EsaD) that inhibit the growth of competing *Staphylococcus* strains [53,54]. The presence of a complete T7SS in our MSSA isolates can suggest their capacity to actively compete for ecological niches. In contrast, the loss of Module 2 in MRSA may be explained by an evolutionary fitness trade-off. Expression of the SCCmec cassette and maintenance of antibiotic resistance impose a substantial metabolic burden on the cell [39]. The absence of Module 2 in our MRSA isolates could potentially be related to metabolic optimization under antibiotic pressure. Our findings highlight two different genomic organization patterns among the isolates: one characterized by a complete T7SS in MSSA and another by SCCmec-mediated resistance in MRSA. Whether these patterns represent stable regional adaptation strategies requires further investigation with a larger and more diverse set of isolates.

The detection of lukF-PV in two of our seven clinical pneumonia isolates (No. 7 and No. 13), as well as in one comparative isolate (OTT1-2022), represents an interesting observation in this cohort. The presence of this toxin in the hypervirulent BORSA strain (No. 13) is particularly significant, as PVL is a key determinant often associated with severe clinical outcomes [55,56,57]. The presence of these genes in our cohort highlights that high virulence potential is not restricted to MRSA strains in the Karaganda region. Notably, the additional protease hysA was identified exclusively in MSSA and BORSA isolates, aligning with the hypothesis of an inverse relationship between antibiotic resistance and virulence gene content. For our results, this means that clinical severity in the region may be driven by these sporadic virulence factors, even in phenotypically susceptible strains.

Despite the presence of multiple virulence-associated genes, all patients in this cohort had favorable clinical outcomes and were discharged after treatment. The favorable clinical outcomes likely result from prompt empirical treatment and the absence of severe comorbidities. PVL-negative *S. aureus* infections are typically associated with older patients and, in pneumonia, with higher short-term survival compared to PVL-positive necrotizing cases [58,59], which may have contributed to the 100% recovery observed in our cohort.

Our study has limitations. As a pilot investigation, the sample size of *S. aureus*-positive pneumonia cases is modest, and the single time-point, single-region design limits generalizability. The lack of transcriptional data for key genes (*mecA*, *femA*, *pbp2*) and detailed patient treatment histories limits the ability to establish direct causal links between genotype, expression, and clinical outcome. Another limitation is that the absence of the *blaZ*, *mecA*, and *mecC* genes was inferred solely from WGS data and was not confirmed by PCR. Future studies should incorporate PCR-based detection of common resistance determinants to rule out potential bioinformatic artifacts arising from fragmented assemblies.

Thus, our study provides an initial insight into the genomic diversity of *S. aureus* in patients with pneumonia in the Karaganda region, indicating that resistance profiles may extend beyond the simple MSSA/MRSA classification. In this cohort, discrepancies between phenotypic and genotypic resistance profiles were observed, including the presence of cryptic MRSA (mecA+) and BORSA isolates. These findings raise the possibility that traditional surveillance approaches could underestimate the true AMR threat. Consequently, the integration of molecular detection of *mecA*, particularly in cases of severe pneumonia or β-lactam treatment failure, may improve classification accuracy and support more informed therapeutic decision-making. Our findings provide a preliminary genomic baseline for pneumonia-associated *S. aureus* in the region and underscore the value of implementing large-scale WGS-based surveillance to inform antimicrobial stewardship and infection control programs in Kazakhstan.

## 4. Materials and Methods

### 4.1. Study Design and Sample Collection

This laboratory-based cross-sectional study was conducted from 2022 to 2023 at the Kazakhstan–Japan Innovation Center of the Kazakh National Agrarian Research University (Almaty, Kazakhstan) and the Research Laboratory of Karaganda Medical University (Karaganda, Kazakhstan), utilizing a convenience sampling strategy based on availability.

We collected 48 sputum samples from adult patients aged between 18 and 90 years with a clinical diagnosis of community-acquired pneumonia (CAP) at three medical institutions in Karaganda: the clinic of Karaganda Medical University (*n* = 28), the Hematology Center (*n* = 17), and the Cardiology Center (*n* = 3). Samples were obtained within the first 48 h of hospitalization to minimize the inclusion of hospital-acquired pneumonia. Exclusion criteria included age under 18 years and pregnancy. The study protocol was approved by the Ethics Committee of the Kazakh National Agrarian Research University and written informed consent was obtained from all participants prior to sample collection.

Sputum samples were collected in accordance with World Health Organization (WHO) and U.S. Centers for Disease Control and Prevention (CDC) guidelines [60,61]. Samples were transported to the laboratory under appropriate conditions and stored at –80 °C until processing.

### 4.2. Bacterial Identification and Antimicrobial Susceptibility Testing

Sputum samples were processed for bacterial culture using standard microbiological methods [62]. Isolates were identified to the species level by matrix-assisted laser desorption/ionization–time-of-flight mass spectrometry (MALDI-TOF MS) using the Microflex LT system and MALDI Biotyper Compass 4.1.80 software (Bruker Daltonics, Bremen, Germany) [63].

Antimicrobial susceptibility testing was carried out using the disk diffusion method on Mueller–Hinton agar (ReadyMED^®^ RDM-MHA-01, Chaitanya Agro Biotech Pvt. Ltd., Malkapur, India) according to the guidelines of the European Committee on Antimicrobial Susceptibility Testing (EUCAST v11.0) [64]. Quality control strains included *Escherichia coli* ATCC^®^ 25922, *E. coli* ATCC^®^ 35218, and *Pseudomonas aeruginosa* ATCC^®^ 27853. Susceptibility results were interpreted following EUCAST v11.0 [64], clinical breakpoints for all antibiotics, except colistin, which was interpreted using CLSI M100 ED33 breakpoints. The antimicrobial susceptibility data were recorded and processed using the AMRmap web-based database [65].

### 4.3. DNA Extraction of S. aureus

Genomic DNA was extracted using the PureLink Microbiome DNA Purification Kit (Invitrogen, Carlsbad, CA, USA) following the manufacturer’s instructions. DNA quality was assessed using a Nanodrop-2000 (Thermo Fisher Scientific, Wilmington, DE, USA) spectrophotometer, and DNA concentration was measured with a Qubit 3.0 fluorometer using the Qubit 1x dsDNA HS Assay Kit (Invitrogen, Carlsbad, CA, USA).

### 4.4. Library Preparation and Whole-Genome Sequencing

Sequencing libraries were prepared from 50 ng of genomic DNA using the Ion Xpress™ Plus Fragment Library Kit (ThermoFisher, Waltham, MA, USA) according to the manufacturer’s protocol. Libraries were quantified with the Ion Library TaqMan^®^ Quantitation Kit (ThermoFisher, Waltham, MA, USA). Template preparation and sequencing were performed on the Ion Chef and Ion Torrent S5 systems using the Ion 540 Chef Kit (ThermoFisher, Carlsbad, CA, USA) and Ion 540 Chip Kit (ThermoFisher, Carlsbad, CA, USA).

### 4.5. Genomic Assembly and Annotation

Raw sequencing reads were quality-trimmed and filtered using FaQCs v. 2.12 tool [66]. De novo genome assembly was performed with SPAdes v.3.15.5 [67]. Assembly quality was evaluated using QUAST v.5.2.0 [68]. Genome annotation was carried out with PROKKA v.1.14.6 [69]. Multilocus sequence typing (MLST) was performed using the mlst software “https://github.com/tseemann/mlst (accessed on 18 December 2025)” using the *S. aureus* MLST scheme described by Enright et al. (2000) [70].

### 4.6. AMR and Virulence Gene Identification

Antimicrobial resistance and virulence gene profiling was performed on clinical and comparative isolates. The dataset consisted of clinical isolates obtained in this study and additional local comparative genomes retrieved from the NCBI database (OTT1-2021, WND1-2021, OTT1-2022). Laboratory reference strains (NCTC 8325 and MOK063), along with the *S. epidermidis* ATCC 14990 outgroup, were included exclusively for phylogenetic tree reconstruction and were not considered in the analyses of antimicrobial resistance and virulence determinants. Genes associated with AMR were identified using the Comprehensive Antibiotic Resistance Database (CARD) through ABRicate (https://github.com/tseemann/abricate, accessed 20 September 2025) [71]. Virulence factor genes were identified using the Virulence Factor Database (VFDB) [72].

### 4.7. Phylogenetic Analysis

A core genome alignment was generated from the seven study isolates and five geographically relevant *S. aureus* genomes retrieved from the NCBI database (accession numbers: CP082813.1, NZ_CP082815.1, CP102945, CP000253, CP029629.1), and *S. epidermidis* ATCC 14990 (GenBank: GCA_006094375.1), which was included as an outgroup. The pangenome was analyzed using Roary v3.13.0 with a 95% BLASTp v2.16.0+ identity threshold, defining the core genome as genes present in ≥99% of isolates [73]. A maximum-likelihood phylogenetic tree was constructed from the core genome alignment using IQ-TREE v3.0.1 (https://github.com/iqtree/iqtree3, accessed 18 December 2025). The tree was visualized and annotated using the ggtree package in RStudio 2026.01.0+392.

## 5. Conclusions

This study reports the presence of the virulent and resistant ST22-CC22 *S. aureus* clone among the seven clinical isolates within this pneumonia cohort in Karaganda, Kazakhstan. We identify a potential diagnostic challenge posed by the co-circulation of cryptic MRSA (*mecA*+, phenotypically susceptible) and hypervirulent BORSA (*mecA*−, phenotypically resistant) strains. While the analysis was based on a limited cohort typical of pilot whole-genome investigations, these results provide preliminary genomic insight into the local population structure. These findings reveal a more complex local epidemiology than previously recognized and underscore the importance of expanding genomic surveillance within national AMR stewardship and infection control programs to better understand the regional spread of these strains and guide effective clinical management.

## Figures and Tables

**Table 1:** Clinical *S. aureus* isolates from pneumonia patients and their institutional distribution.

No.	Isolate_ID	Gender	Age	Outcome	Institution *	Department	Collection Date	Specimen
7	57537	F	75	Recovery	HEM	Hematology (HO)	15 November 2022	sputum
10	57595	F	60	Recovery	HEM	Hematology (HO)	22 November 2022	sputum
13	57706	M	64	Recovery	UNC	Surgery (SUR)	1 December 2022	sputum
20	58728	F	47	Recovery	UNC	Surgery (SUR)	13 March 2023	sputum
47	60754	F	25	Recovery	UNC	Pulmonology (PUL)	29 September 2023	sputum
54	60984	M	71	Recovery	UNC	Surgery (SUR)	26 October 2023	sputum
58	61130	F	73	Recovery	HEM	Hematology (HO)	9 November 2023	sputum

* HEM: Hematology Center; UNC: University Clinic of Karaganda Medical University.

## Data Availability

The raw data have been submitted to NCBI. The project accession number is PRJNA1165446.

## Supplementary Materials

The following supporting information can be downloaded at: https://www.mdpi.com/article/10.3390/antibiotics15050431/s1, Table S1: Genome assembly statistics; Table S2: AMR genes; Table S3: virulence factor genes.
